# Pathways leading to prevention of fatal and non-fatal cardiovascular disease: An interaction model on 15 years population-based cohort study

**DOI:** 10.1186/s12944-020-01375-8

**Published:** 2020-09-05

**Authors:** Najmeh Shakibaei, Razieh Hassannejad, Noushin Mohammadifard, Hamid Reza Marateb, Marjan Mansourian, Miguel Angel Mañanas, Nizal Sarrafzadegan

**Affiliations:** 1 School of Public Health, Isfahan University of Medical Sciences, Isfahan, Iran; 2 Interventional Cardiology Research Center, Cardiovascular Research Institute, Isfahan University of Medical Sciences, Isfahan, Iran; 3 Hypertension Research Center, Cardiovascular Research Institute, Isfahan University of Medical Sciences, Isfahan, Iran; 4 Biomedical Engineering Department, Engineering Faculty, University of Isfahan, Isfahan, Iran; 5 Department of Automatic Control, Biomedical Engineering Research Center, Universitat Politècnica de Catalunya, BarcelonaTech (UPC), Barcelona, Spain; 6 Pediatric Cardiovascular Research Center, Cardiovascular Research Institute, Isfahan University of Medical Sciences, Isfahan, Iran; 7 Biomedical Research Networking Center in Bioengineering, Biomaterialsand Nanomedicine (CIBER-BBN), Barcelona, Spain; 8 Isfahan Cardiovascular Research Center, Cardiovascular Research Institute, Isfahan University of Medical Sciences, Isfahan, Iran; 9 School of Population and Public Health, Faculty of Medicine, University of British Columbia, Vancouver, Canada

**Keywords:** Acute coronary syndrome, Bayesian approach, Cardiovascular disease, Stroke, Structural equation models

## Abstract

**Background:**

A comprehensive study on the interaction of cardiovascular disease (CVD) risk factors is critical to prevent cardiovascular events. The main focus of this study is thus to understand direct and indirect relationships between different CVD risk factors.

**Methods:**

A longitudinal data on adults aged ≥35 years, who were free of CVD at baseline, were used in this study. The endpoints were CVD events, whereas their measurements were demographic, lifestyle components, socio-economics, anthropometric measures, laboratory findings, quality of life status, and psychological factors. A Bayesian structural equation modelling was used to determine the relationships among 21 relevant factors associated with total CVD, stroke, acute coronary syndrome (ACS), and fatal CVDs.

**Results:**

In this study, a total of 3161 individuals with complete information were involved in the study. A total of 407 CVD events, with an average age of 54.77(10.66) years, occurred during follow-up. The causal associations between six latent variables were identified in the causal network for fatal and non-fatal CVDs. Lipid profile, with the coefficient of 0.26 (0.01), influenced the occurrence of CVD events as the most critical factor, while it was indirectly mediated through risky behaviours and comorbidities. Lipid profile at baseline was influenced by a wide range of other protective factors, such as quality of life and healthy lifestyle components.

**Conclusions:**

Analysing a causal network of risk factors revealed the flow of information in direct and indirect paths. It also determined predictors and demonstrated the utility of integrating multi-factor data in a complex framework to identify novel preventable pathways to reduce the risk of CVDs.

## Background

Cardiovascular disease (CVD), which is responsible for 17.9 million deaths, has been identified as the world’s leading cause of death [1, 2]. The World Health Organization (WHO) estimates that this number increases to 23.6 million by 2030 [3]. In low- and middle-income countries, more than three-quarters of deaths occur due to CVD, which results in the imposition of 80% of the global burden of CVD. Iran has a similar situation in which the burden of CVD will be more significant in the upcoming years [4, 5]. However, most of the risk factors of this non-communicable disease (NCD) are “preventable” [6]. Therefore, the global strategy is to reduce the early incidence of CVD. The prevention of CVD can be four times more effective than subsequent measures [7]. Identifying relevant risk factors is the best way to correct the risky behaviours that make a person vulnerable to disease [8]. The interaction of irreversible factors (e.g., age, sex, family history of early CVD and race), behavioural factors (e.g., physical activity, smoking, and diet), and physiological risk factors (e.g., diabetes mellitus (DM), blood pressure (BP), obesity, and dyslipidaemia) may cause CVDs [9–15].

Many studies examined CVD risk factors [16–21]. However, they cannot usually detect causal pathways between the associated factors and CVD. Some studies investigated cardiovascular risk factors using structural equation modelling [17–21]. Among which, Azizi et al. studied the effect of emotional distress, physical activity, and body mass index (BMI) on the link between CVD and short sleep duration (< 7 h per day) [17]. Scott-Storey et al. studied the severity of women’s abuse that might affect women’s risk of CVD providing preliminary evidence to support many hypothesised pathways using the severity of abuse [18]. Fong and Ho used Bayesian structural equation modelling (BSEM) to identify the association between depression scale, and the hospital anxiety a sample of 312 Chinese patients. It showed that the two-factor structure represents the appropriate scale of hospital depression and anxiety in clinical practice [19]. Kerkhof et al. presented SEM with different pathways indicating fat mass is influenced by atherosclerosis risk factors at the age of 21 years [20]. Goong et al. indicated that health behaviour modification performance could be described by health belief, social support, and changing the knowledge and attitude about health using the SEM in patients with CVDs [21].

Many CVD factors have been identified in previous studies, but it is not clear whether these factors influence CVDs directly or indirectly. Meanwhile, CVDs are chronic diseases influenced by various factors. Therefore, it is essential to study the multidimensional factors at the same time leading to CVD. To the best of our knowledge, no such multidimensional study approaches have been applied to the problem of different lifestyle components, laboratory factors, and comorbidities, at the same time, with indirect and direct effects. In this study, a series of BSEMs to examine the associations between a large number of risk factors and risk for total CVDs and its different categories were developed. By understanding the contribution of these factors leading CVDs, the findings of this study could be of great help for healthcare professionals in recognising strong predictors for applying effective strategies for CVDs prevention.

## Materials and methods

### Study population

Isfahan cohort study (ICS), a population-based study, has been performed by Isfahan Cardiovascular Research Center (ICRC), a WHO-collaborating centre (https://apps.who.int/whocc/Detail.aspx?9Dsb+Z/ZGeLpVAygEBL+Hg==). It was designed to identify the effect of different risk factors on the incidence of CVD in a representative sample from the centre of Iran, including Isfahan, Arak, and Najafabad. Participants were selected using multistage cluster random sampling based on gender, age, and residence status (urban/rural) distribution. They were recruited from 2001 and were followed up every 2 years for major cardiovascular events and every 5-years for measuring risk factors. Inclusion criteria included (1) being at least 35 years old without CVD and mental retardation diseases, (2) having Iranian nationality, (3) living in one of the above three cities for at least 6 months, and (4) not being pregnant [22]. This study is the secondary analysis of the ICS, which had the power of 90% to detect new CVD cases [22].

### Risk factors measurements

Trained health professionals collected demographic, behavioural, socioeconomic characteristics, smoking, nutrition, and physical activity status. Smoking status was classified as non-smokers, ex-smokers, and smokers. Smokers were who smoked at least one cigarette a day while individuals who smoked before but did not smoke at the time of the interview (i.e., those who replied “no” to the question “are you smoking now?”) were defined as ex-smokers. Fasting blood samples (10 ml) were taken from participants to measure triglycerides (TG), low*-density lipoproteins* (LDL-C), *high-density lipoproteins* (HDL-C), and total cholesterol (Tcho). Diabetes was considered if fast blood sugar (FBS) ≥ 126 mg/dL or taking hypoglycaemic agents [23]. Dyslipidaemia was defined if HDL-C ≤ 50 mg/dL in women, and HDL-C ≤ 40 mg/dL in men, Tcho ≥200 mg/dL, TG ≥ 150 mg/dL, and LDL-C ≥ 130 mg/dL. The anthropometric parameters weight, height, waist circumference (WC), and hip circumference (HC) were measured by standard protocols. Obesity was considered as body mass index (BMI) ≥ 30 kg / m^2^. Abdominal obesity was defined as HC ≥85.5 cm in women and HC ≥91.5 cm in men, and high WC was defined as WC ≥94 cm in men or WC ≥ 80 cm in women [24, 25]. Trained professionals measured systolic and diastolic blood pressure with standard protocol. According to the WHO definition, people with diastolic blood pressure (DBP) ≥ 90 or systolic blood pressure (SBP) ≥ 140 mmHg or those using hypertensive drug users were considered as hypertensive patients [26].

### Instruments and questionnaires

Dietary behaviours were assessed by a validated food frequency questionnaire (FFQ) [27]. Factor analysis was used to identify four main dietary patterns. The Mediterranean diet pattern was considered as the CVD mortality protective regime (i.e., healthy diet behaviour), while the other diet patterns were considered as an unhealthy diet behaviour [14]. A validated international physical activity questionnaire (IPAQ) was used to assess physical activity [28]. Depression and anxiety were detected using A general health questionnaire (GHQ-28) [29].

The quality of life was assessed using the WHO quality of life questionnaire (WHOQOL-BREF), including 26 questions, four subscales, and an overall score. Four areas of QOL were environmental health, social relations, mental, and physical health. Transportation, physical environment (weather, traffic, noise, and pollution), home environment, quality of social care, security, health and access, freedom, and financial resources were considered for environmental health. Sexual activities, social support, and personal relationships were considered for social relations. Mental health, on the other hand, consisted of focus, memory, learning, thinking, personal beliefs, spirituality or religion, self-esteem, positive feelings, and negative emotions. Work capacity, rest, sleep, discomfort, pain, mobility, fatigue, energy, medical assistance, drug dependency, and activities of daily living were considered for physical health [30–33].

### Outcomes

Total CVD included significant events such as fatal and non-fatal myocardial infarction (MI), unstable angina (UA), sudden cardiac death (SCD), and fatal and non-fatal stroke. Two separate groups of specialists, each comprising four neurologists and cardiologists, reviewed all patient records (original questionnaires, secondary interviews, medical records, verbal autopsy, or death certificates). Then, the final decision was made on cardiovascular events. Ischemic heart disease (IHD) included MI, sudden cardiac death, and definitive or probable UA. The diagnosis of stroke was based on the WHO definition as a permanent neurological disorder for at least 24 h. Acute coronary syndrome included all events of CVDs except stroke and sudden cardiac death. Fatal CVD included fatal strokes, fatal MI, and sudden cardiac death [22]. Total CVD, stroke, ACS, and fatal CVD were separately considered in different risk factors’ causal networks.

### Statistical analysis

Continuous variables were represented by the mean and standard deviation (SD), while a number and percentage were used for the categorical variables. Comparisons in groups were obtained using logistic regression. Analyses were performed with STATA 14 (StataCorp. 2015. Stata Statistical Software: Release 14. College Station, TX: StataCorp LP.) and open BUGS version 3.2.3. A pairwise correlation test was used to produce the correlation matrix for each of the variables known or hypothesised to influence the CVD in adults.

### Modelling description

SEM with a mixture of continuous and dichotomous variables was used to identify factors associated with fatal CVD, ACS, stroke, and total CVD. It was used to measure latent concepts such as healthy, risky behaviour or quality of life, which were not directly measured, as well as to explore the pattern of both direct and indirect relationships between the factors associated with the CVDs [34]. Due to a large number of explanatory variables, the Bayesian SEM was applied. In a Bayesian method, which is a broad approach to deal with complex situations, the useful prior information is used, and reliable results with small or medium sample size can be presented. The Bayesian approach is also used as an attractive method to generalise basic SEMs such as nonlinear, multilevel, and longitudinal SEMs [35]. We implement Markov Monte Carlo Chain Methods (MCMC), and observations were obtained by Gibbs sampling on conditional density in open BUGs (Supplementary Materials 1, and 2, Additional file 1).

**Fig. 1:**
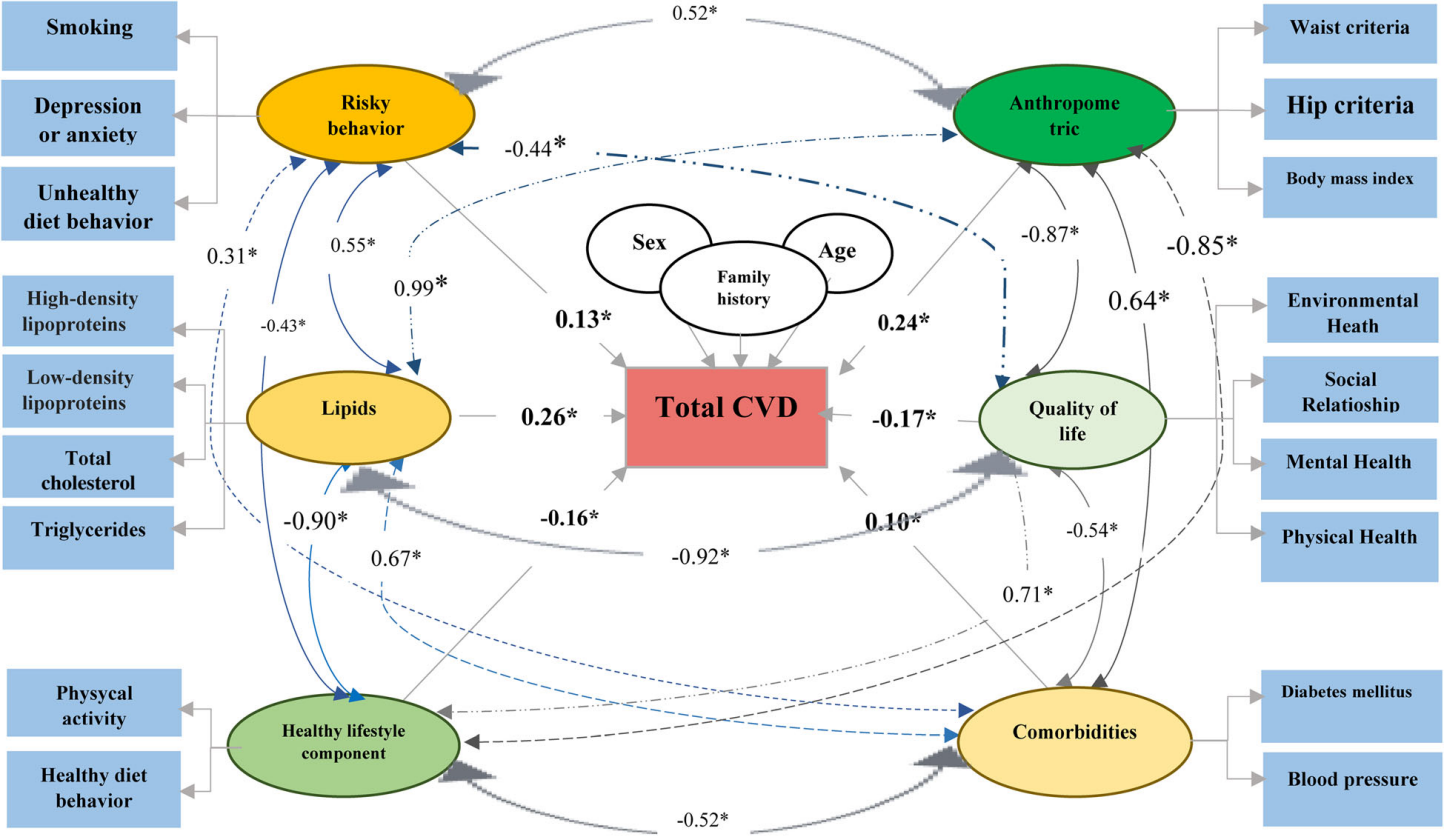
BSEM the mediating effects of factor associated with total CVD in the Isfahan Cohort Study

As shown in Fig. 1, the latent variables considered in this study were lipids, anthropometric, risky behaviour, comorbidities, quality of life, and healthy lifestyle component. Figure 1 contains 21 indicator variables and 6 exogenous latent variables and one endogenous latent variable.

The structural equation for CVD is shown as follows:

1 $$ \mathrm{Lipids}+{\upgamma}_2\times \mathrm{Anthropometric}+{\upgamma}_3\times \mathrm{Risky}\ \mathrm{behavior}+{\upgamma}_4\times \mathrm{Comorbidities}+{\upgamma}_5\times \mathrm{Quality}\ \mathrm{of}\ \mathrm{life}+{\upgamma}_6\times \mathrm{Healthy}\ \mathrm{life}\mathrm{style}\ \mathrm{component}+{\updelta}_{\mathrm{i}}; $$

where γ_*i*_ is the impact coefficient of latent variables and δ_i_ is the random vector of the residuals.

## Result

### Descriptive statistics

Factor analysis was used to obtain a nutritional pattern. Four main dietary patterns were identified, including: “fast food”, “animal fat”, “Mediterranean”, and “western”. The “fast food diet” contained lots of carbonated drinks, sweets, pizzas, sausages, and burgers, while the “animal fat diet” was described by a high intake of meat, butter, whole milk, and cream. The consumption of olive oil, vegetables, fish, poultry, fruits, and non-hydrogenated vegetable oils (NHVO) were associated with the Mediterranean diet. The “western diet” included rice, pickles, red meat, hydrogenated vegetable oils, legumes, potatoes, and fried foods (Supplementary Table 1, Additional file 1).

In this study, from a total number of 3161 people, 180(0.05%) men and 227(0.07%) women with total CVD were observed. CVD-related risk factors, including lipids (LDL-C, TG, Tcho), anthropometric (HC, WC, BMI), risky behaviour (depression or anxiety, unhealthy diet behaviour, smoking), and comorbidities (BP, DM), were higher than those without CVD (Tables 1, and 2). The CVD group’s healthy diet and physical activity, on the other hand, were lower than non-CVD subjects. It is also worth noting that a higher score in Tables 1, and 2 shows a lower quality of life. Patients with CVD had a lower quality of life compared with others.

**Table 1:** Characteristics of study participants for CVD and Stroke

Characteristics	CVD		*P*-value	Stroke		*P*-value
	No *N* = 2754	Yes *N* = 407		No *N* = 3084	Yes *N* = 77	
Demographic Variables						
Age (years)	48.39 (9.96)	54.77 (10.66)	0.00	49.04 (10.19)	56.08 (11.33)	0.18
Sex			0.01			0.52
Man	1438 (52.21%)	180 (44.22%)		1583 (51.3%)	35 (45.5%)	
Woman	1316 (47.79%)	227 (55.78%)		1501 (48.7%)	42 (54.5%)	
Family History of CVD						0.15
Yes	152 (5.50%)	34 (8.4%)		179 (5.8%)	7 (9.1%)	
No	2602 (94.50%)	373 (91.6%)		2905 (94.2%)	70 (90.9%)	
Lipids						
HDL (mg/dL)	46.88 (10.29)	46.71 (10.57)	0.76	46.85 (10.32)	47.7 (10.67)	0.85
LDL (mg/dL)	127.66 (42.89)	135.81 (44.87)	0.00	128.67 (43.23)	130.3 (43.33)	0.74
TG (mg/dL)	186.58 (99.77)	222.22 (117.27)	0.00	190.46 (102.34)	218.99 (119.57)	0.04
TChol (mg/dL)	211.86 (51.37)	226.97 (55.06)	0.00	213.62 (52.06)	221.1 8 (55.36)	0.20
Anthropometric						
HC (cm)	101.99 (9.51)	102.63 (9.64)	0.20	102.03 (9.53)	103.95 (9.41)	0.08
WC (cm)	94.52 (11.90)	97.86 (12.43)	0.00	94.87 (12.00)	98.27 (12.27)	0.19
BMI (kg / m^2^)	26.82 (4.33)	27.41 (4.64)	0.01	26.87 (4.35)	28.08 (5.10)	0.04
Risky behaviour						
Depression or anxiety	1.97 (2.19)	2.44 (2.69)	0.00	2.03 (2.25)	2.44 (2.84)	0.21
Unhealthy diet behaviour	2.12 (2.46)	1.71 (2.13)	0.00	2.08 (2.42)	1.81 (2.54)	0.36
Smoking Status			0.00			0.01
Smoker	414 (15.03%)	81 (19.90%)		477 (15.5%)	18 (23.4%)	
Ex-Smoker	154 (5.59%)	32 (7.86%)		180 (5.8%)	6 (7.8%)	
Non-Smoker	2186 (79.37%)	294 (72.23%)		2427 (78. 7%)	53 (68.8%)	
Comorbidities						
BP			0.46			0.00
Yes	660 (24%)	179 (44%)		796 (25.8%)	43 (55.8%)	
No	2094 (76%)	228 (56%)		2288 (74.2%)	34 (44.2%)	
DM			0.00			0.60
Yes	227 (8.2%)	97 (23.8%)		305 (9.9%)	19 (24.7%)	
No	2527 (91.8%)	310 (76.2%)		2779 (90.1%)	58 (75.3%)	
Quality of life						
Environmental Health	32.76 (17.02)	39.47 (16.67)	0.00	33.37 (17.07)	43.48 (16.67)	0.00
Social Relationship	33.92 (15.72)	36.40 (16.16)	0.00	34.12 (15.75)	38.87 (17.00)	0.01
Mental Health	28.14 (18.62)	29.88 (19.22)	0.08	28.24 (18.66)	33.49 (19.70)	0.01
Physical Health	34.45 (16.63)	37.00 (16.25)	0.00	34.63 (16.61)	41.00 (14.90)	0.00
Healthy lifestyle component						
Healthy diet	2.33 (3.09)	1.83 (2.41)	0.00	2.28 (3.02)	1.83 (2.70)	0.19
Daily physical activity (METs-min/day)	910.65 (539.56)	839.50 (562.06)	0.01	904.11 (543.70)	796.63 (503.70)	0.08

*Abbreviations*: cardiovascular disease, *HDL* high-density lipoproteins-cholesterol, *LDL* low*-*density lipoproteins –cholesterol, *TG* triglycerides, *TC* total cholesterol, *HC* hip circumference, *WC Waist* circumference, *BMI* Body mass index, *BP* blood pressure, *DM* diabetes mellitus

**Table 2:** Characteristics of study participants for models (Acute coronary disease and Fatal CVD)

Characteristics	ACS		*P*-value	Fatal CVD		*P-value*
	No *N* = 2846	Yes *N* = 315		No *N* = 2979	Yes *N* = 182	
Demographic variables						
Age (years)	48.39 (9.9)	53.9 (10.3)	0.00	48.3 (9.9)	55.8 (11.5)	0.00
Sex						
Man	1478 (51.53%)	140 (44.44%)	0.00	1550 (52.03%)	69 (37.91%)	0.00
Woman	1368 (48.47%)	175 (55.56%)		1429 (47.97%)	113 (62.09%)	
Family History of CVD						
Yes	174 (6.11%)	27 (8.60%)	0.00	264 (8.80%)	11 (6.04%)	0.56
No	2672 (93.89%)	288 (91.40%)		2715 (91.20%)	171 (93.96%)	
Lipids						
HDL (mg/dL)	46.88 (10.29)	46.65 (10.54)	0.7	46.8 (10.2)	46.0 (10.2)	0.29
LDL (mg/dL)	127.66 (42.89)	138.01 (45.6)	0.00	127.6 (42.8)	133.6 (45.3)	0.06
TG (mg/dL)	186.5 (99.77)	223.8 (117.8)	0.00	186.5 (99.7)	217 (113.3)	0.00
TChol (mg/dL)	211.86 (51.3)	229.4 (56.1)	0.00	211.8 (51.3)	223.2 (53.8)	0.00
Anthropometric						
HC (cm)	101.9 (9.5)	102.5 (9.7)	0.36	101.9 (9.5)	101.8 (0.2)	0.85
WC (cm)	94.5 (11.9)	97.81 (12.5)	0.00	94.5 (11.9)	97.0 (13.1)	0.01
BMI (kg / m^2^)	26.82 (4.33)	27.34 (11.94)	0.04	26.8 (4.33)	27.2 (4.8)	0.15
Risky behaviour						
depression or anxiety	1.97 (2.1)	2.4 (2.6)	0.00	1.9 (2.1)	2.5 (2.7)	0.01
unhealthy diet behaviour	2.1 (2.4)	1.7 (2.0)	0.00	2.1 (2.4)	1.7 (2.0)	0.02
Smoking Status			0.01			0.00
Smoker	434 (15.25%)	61 (19.37%)		489 (16.41%)	45 (24.73%)	
Ex-Smoker	161 (5.67%)	25 (7.94%)		229 (7.68%)	12 (6.60%)	
Non-Smoker	2251 (79.08%)	229 (72.69%)		2261 (75.91%)	125 (68.67%)	
Comorbidities						
BP						
Yes	709 (24.91%)	132 (41.90%)	0.63	772 (25.91%)	81 (44.51%)	0.05
No	2137 (75.08%)	183 (58.10%)		2207 (74.09%)	101 (55.49%)	
DM						
Yes	255 (8.96%)	74 (23.50%)	0.00	339 (11.37%)	45 (24.73%)	0.001
No	2591 (91.04%)	241 (76.50%)		2640 (88.63%)	137 (75.27%)	
Quality of life						
Environmental Health	32.7 (17.0)	38.0 (16.4)	0.00	32.7 (17.0)	40.6 (16.5)	0.00
Social Relationship	33.9 (15.7)	35.1 (15.8)	0.18	33.9 (15.7)	36.9 (15.9)	0.01
Mental Health	28.1 (18.6)	28.3 (19.0)	0.84	28.1 (18.6)	31.4 (18.5)	0.02
Physical Health	34.4 (16.6)	35.5 (16.5)	0.2	34.4 (16.6)	38.3 (15.9)	0.00
Healthy lifestyle component						
Healthy diet	2.3 (3.0)	1.8 (2.3)	0.00	2.3 (3.0)	1.9 (2.4)	0.13
Daily physical activity (METs-min/day)	910.6 (539.5)	851.0 (571.3)	0.006	910.6 (539.5)	828.3 (521.1)	0.04

*Abbreviations*: *CVD* cardiovascular disease, *ACS* Acute coronary artery syndrome, *HDL high-density lipoproteins-*cholestero*l*, *LDL* low*-density lipoproteins* –cholesterol, *TG* triglycerides, *TC* total cholesterol, *HC* hip circumference, *WC Waist* circumference, *BMI* Body mass index, *BP* blood pressure, *DM* diabetes mellitus

The lipid profiles (HDL-C, LDL-C, total Cho) have the highest impact on CVD. Other significant variables were anthropometric indices (WC, and HC, and BMI), risky behaviour (depression or anxiety, unhealthy diet behaviour, and smoking status), comorbidities (DM, BP), quality of life (environmental health, social relationship, mental health, physical health), and healthy lifestyle components (physical activity and healthy diet behaviour) (Fig. 1). Thus, the higher the level of lipid profiles, the higher the risk of CVDs. Also, the association between higher quality of life and a healthy lifestyle with CVDs incidence was significantly negative.

Applying the BSEM, a causal network is plotted using different exploratory factors, twenty-one measurements of CVD risk factors at baseline, including cardiovascular risk factors. Models obtained in this study classified these variables into six latent variables (Table 3). Also, in line with this study, models for stroke, ACS, and fatal CVD (Tables 4 and 5) were developed. The most important finding of this study based on the comparison of three models of stroke, acute coronary syndrome, and fatal CVD, is the significant effect of risky behaviour on these subtypes of CVD (Stroke (0.14), ACS (0.20), Fatal CVD (0.11)). Other direct and indirect relationships of all three models are presented in Tables 4 and 5. It should be noted that all relationships were significant except for a family history of CVD.

**Table 3:** Estimated direct, indirect effects related factor for CVD

Type of effect							
Direct effect				Indirect effect			
	Coefficient	SD	95% CI		Coefficient	SD	95% CI
Age- > total CVD	0.002	0.0008	(0.001,0.004)	**Lipids- >** HDL	1		
				**Lipids- >** LDL	0.25	0.007	(0.22,0.26)
				**Lipids- >** Tgd	0.84	0.02	(0.81, 0.88)
				**Lipids- >** Cho	0.47	0.01	(0.44,0.50)
Family history - > total CVD	0.000	0.0006	(−0.001,0.0009)	**Anthropometric- >** HC	1		
				**Anthropometric- >** WC	0.78	0.006	(0.77,0.80)
				**Anthropometric- >** BMI	0.10	0.01	(0.08,0.11)
Sex - > total CVD	0.004	0.001	(0.007,0.012)	**Unhealthy life style- >** smoking	1		
				**Risky behaviour - >** depression and anxiety	0.79	0.1	(0.42,1.22)
				**Risky behaviour - >** unhealthy diet behaviour	− 0.57	0.2	(− 0.99, − 0.16)
Lipids - > total CVD	0.26	0.01	(0.26,0.28)	**Comorbidities- >** DM	1		
				**Comorbidities- >** BP	0.74	0.006	(0.73, 0.75)
Anthropometric - > total CVD	0.24	0.01	(0.22,0.26)	**Quality of life- >** Environmental Heath	1		
				**Quality of life- >** Social Relationship	0.45	0.004	(0.44,0.47)
				**Quality of life- >** Mental Health	0.52	0.005	(0.51,0.54)
				**Quality of life- >** Physical Health	0.53	0.004	(0.51,0.54)
risky behaviour- > total CVD	0.13	0.03	(0.10,0.14)	**Healthy lifestyle component- >** physical activity	1		
				**Healthy lifestyle component- >** Healthy diet behaviour	0.99	0.01	(0.95,1.03)
Comorbidities - > total CVD - > CVD	0.10	0.001	(0.08, 0.11)	**Lipids** < −> Anthropometric	0.99	0.15	(0.84,1.49)
				**Lipids** < −> Risky behaviour	0.55	0.13	(0.22,0.80)
				**Lipids** < −> Comorbidities	0.67	0.08	(0.53,0.89)
				**Lipids** < −> Quality of life	−0.92	0.12	(−1.18, −0.71)
				**Lipids** < −>healthy lifestyle component	−0.90	0.12	(−1.16, − 0.70)
Quality of life - > total CVD	− 0.17	0.01	(− 0.18, − 0.15)	**Anthropometric** < −> Risky behaviour	0.52	0.12	(0.22,0.75)
				**Anthropometric** < −> Comorbidities	0.64	0.09	(0.50, 0.85)
				**Anthropometric** < −> Quality of life	−0.87	0.12	(−1.12, − 0.67)
				**Anthropometric** < −> healthy lifestyle component	−0.85	0.11	(− 1.11, − 0.67)
Healthy lifestyle - > total CVD	− 0.16	0.008	(− 0.17, − 0.14)	**Risky behaviour** < −> Comorbidities	0.315	0.07	(0.13,0.45)
				**Risky behaviour** < −> Quality of life	−0.44	0.10	(−0.63, − 0.18)
				**Risky behaviour** < −> healthy lifestyle component	−0.43	0.10	(−0.61, − 0.18)
				**Comorbidities** <− > Quality of life	−0.54	0.07	(−0.72, − 0.43)
				**Comorbidities** <− > healthy lifestyle component	−0.52	0.07	(−0.71, − 0.42)
				**Quality of life** < −> healthy lifestyle component	0.71	0.09	(0.56,0.92)

*Abbreviations*: cardiovascular disease, *HDL* high-density lipoproteins-cholesterol, *LDL* low-density lipoproteins –cholesterol, *TG* triglycerides, *TC* total cholesterol, *HC* hip circumference, *WC Waist* circumference, *BMI* Body mass index, *BP* blood pressure, *DM* diabetes mellitus

**Table 4:** Estimated direct effects related factor for models (Stroke, ACS, and Fatal CVD)

Models (Direct effect)			
	Stroke Coefficients (SD)	ACS Coefficients (SD)	Fatal CVD Coefficients (SD)
Age->	0.001 (0.00008)	0.002 (0.00005)	0.002 (0.00006)
Family history ->	0.000 (0.002)	0.000 (0.002)	0.00 (0.003)
Sex ->	0.01 (0.003)	0.009 (0.002)	0.009 (0.003)
Lipids ->	0.26 (0.003)	0.24 (0.003)	0.28 (0.003)
Anthropometric ->	0.23 (0.002)	0.23 (0.002)	0.25 (0.002)
Risky behaviour ->	0.14 (0.007)	0.20 (0.02)	0.11 (0.01)
Comorbidities ->	0.10 (0.0003)	0.11 (0.0008)	0.11 (0.0007)
Quality of life ->	−0.16 (0.002)	−0.15 (0.001)	−0.17 (0.001)
Healthy lifestyle component ->	−0.15 (0.001)	−0.15 (0.001)	− 0.17 (0.001)

**Table 5:** Estimated indirect effects related factor for models (Stroke, ACS, and Fatal CVD)

Models (Indirect effect based on β (SD))			
	Stroke Coefficients (SD)	ACS Coefficients (SD)	Fatal CVD Coefficients (SD)
Lipids<− > Anthropometric	0.99 (0.1)	0.98 (0.1)	0.98 (0.1)
Lipids<− > Risky behaviour	0.60 (0.1)	0.65 (0.4)	0.36 (0.2)
Lipids<− > Comorbidities	0.67 (0.08)	0.64 (0.07)	0.72 (0.08)
Lipids<− > Quality of life	−0.91 (0.1)	−0.86 (0.09)	− 0.98 (0.1)
Lipids<− > healthy lifestyle component	− 0.90 (0.1)	−0.85 (0.09)	− 0.98 (0.1)
Anthropometric<−Risky behaviour	0.57 (0.1)	0.62 (0.4)	0.34 (0.2)
Anthropometric<− > Comorbidities	0.63 (0.08)	0.61 (0.07)	0.69 (0.08)
Anthropometric<− > Quality of life	−0.85 (0.1)	−0.82 (0.09)	− 0.93 (0.1)
Anthropometric<− > healthy lifestyle component	−0.84 (0.1)	− 0.81 (0.09)	−0.93 (0.1)
Risky behaviour <− > Comorbidities	0.32 (0.07)	0.37 (0.2)	0.20 (0.1)
Risky behaviour <− > Quality of life	−0.46 (0.1)	−0.51 (0.3)	− 0.28 (0.1)
Risky behaviour <− > healthy lifestyle component	−0.45 (0.1)	− 0.50 (0.3)	−0.28 (0.1)
Comorbidities<− > Quality of life	− 0.53 (0.07)	−0.51 (0.06)	− 0.56 (0.06)
Comorbidities<− > healthy life style	−0.53 (0.07)	− 0.50 (0.06)	−0.57 (0.09)

Convergence statistical test was performed for all parameters, and the Goleman-Robin test statistic was close to one. The Monte Carlo errors were also very low for the entire parameters in the models. Also, the goodness of fit of the models was assessed using Posterior Predictive *P*-value (P.P. *P*-value). Such values were 0.55, 0.54, 0.54, and 0.49 for CVD, stroke, ACS, and Fatal CVD, respectively (Supplementary Material 3 (A, B, and C), Additional file 1).

## Discussion

In this study, BSEM was used to identify both direct and indirect pathways leading to total CVD, ACS, stroke, and fatal CVDs. Such an increased or decreased correlation between the traditional risk factors was related to the influence of the confounding variables while were adjusted by indirect pathways. Although previous studies considered limited risk factors, mostly in binary scale, this study showed preventive pathways with a large number of risk factors in both continuous and binary measurement scales. The Bayesian framework was applied for SEM modelling, which is reliable, especially in small sample size studies. Moreover, the resampling method used in MCMC was used in this study [36].

Many studies were performed in the literature on CVDs. Although the identification of CVD risk factors seems to be relatively comprehensive, the precise and indirect mechanisms of the associated factors underlying CVD remains unclear [37]. It is a critical gap since healthy behaviours lead to both reduced risk and more effective treatments [17, 38]. This is the first study in the field of CVD, to the best of our knowledge, that extensively examined the direct and indirect effects of comprehensive cardiovascular-related factors using BSEM in a population-based cohort platform.

Although the indirect effects of family history of CVD, age, lipid profiles, anthropometric indices, healthy lifestyle, quality of life, high-risk behaviours, having hypertension and high blood sugar on CVDs were not covered in the literature, many studies discussed the direct effect of such factors in which these results are in line with [9, 11, 12, 15, 38–50]. Causal networks evaluate causal relationships among variables beyond partial correlations and thus play a fundamental step in risk prediction. For example, although many studies listed blood pressure as the most critical risk factor for CVD [51–55], this study did not observe such a relationship. According to the results, CVD is mostly influenced by lipid profiles, which is in line with the CVD prevention study in Japan [56] and Brisighella heart study [57].

The proposed model indicated that the quality of life and healthy lifestyle components have the highest impact on lipid profile to prevent CVD. Therefore, individuals need to improve their quality of life indicators. Taking into account the physical health, as the first step, is essential. Indeed, in the absence of physical health, other indicators of quality of life do not improve. Environmental health was the second effective factor in these results. The social relationships with other people and also mental health improvement can play an essential role in CVD prevention [58, 59]. This study also highlighted the health benefits of high physical activity and having a healthy diet. Healthy lifestyle components also have a strong relationship with the quality of life variable. Therefore, considering the modifiable components of a healthy lifestyle, the healthy diet and the proper amount of physical activity (at least 150 min of physical activity per week [60]), are necessary to prevent CVD in adults [60, 61].

A healthy diet was promoted in the literature to prevent CVD [62]. The Mediterranean diet is one of the diet patterns in recent years, focusing on more consumption of plant foods (e.g., seeds, nuts, beans, fruits, and vegetables) and low to moderate amounts of animal food (e.g., red meat). In this diet, chicken, fish, vegetables, fruits, olive oil, NHVO, and fibres are mostly focused. Unhealthy diets, on the other hand, including western, animal fat, and fast food diets are characterised by various food items with high trans fatty acids (TFA), saturated fatty acid (SFA), and less monounsaturated fatty acid (MUFA) and Polyunsaturated fatty acid (PUFA). Such unhealthy diets also included rice, potato, and sweets, which contain high refined carbohydrates increase TG, and reduce HDL-C, as two essential components in the Iranian lipid profile [14, 62–64].

Obesity and overweight are general risk factors of CVD [48]. Due to the close relationship between lipid profiles and anthropometric indices, it is necessary to take into account the improved lifestyle and quality of life to reduce the incidence of CVD. Moreover, risky behaviour endangers health and quality of life and has a positive direct effect on anthropometric indices and lipid profile. Stress, on the other hand, can have immediate cardiovascular consequences, such as heart attack and sudden cardiac arrest, which can occur even in healthy people [65]. The possible role of smoking-related disorders in CVD should not be ignored either. Since stress and smoking are also modifiable factors, people can easily manage these high-risk behaviours to prevent CVDs [55, 66].

Non-communicable Disease Prevention organisations consider the unhealthy diet, physical inactivity, and alcohol use as common causes for stroke, heart disease, diabetes mellitus, cancer, and lung disease [67]. Also, GBD findings suggest that two-thirds of strokes occur among people under the age of 70, so they should no longer be considered as old-age diseases. It has also been reported that stroke is increasing in young and middle-aged adults, possibly due to increased metabolic risk factors such as obesity and diabetes mellitus. The main risk factors for stroke were smoking and high blood pressure. Other risk factors included obesity, high blood cholesterol, and diabetes mellitus [68]. In the proposed model, anthropometric indices, which were more critical than lipid profiles and risky behaviour, play a significant role in stroke.

Researches showed that the following risk factors increase the likelihood of ACS occurrence: old age, diabetes, hypertension, tobacco use, risky behaviour, male gender, and a family history of heart disease [69]. The results, on the other hand, highlighted the impact of risky behaviour that cannot be ignored. Rücker et al. stated that the main risk factors of fatal CVDs were total cholesterol, smoking, and also having higher systolic blood pressure [70]. Current results showed that the lipid profile was the most critical factor for (fatal) CVDs, emphasising the control of lipid profile.

In this study, a reliable statistical approach was used on a representative population of the Eastern and Mediterranean region (EMR), aiming to guide healthcare policymakers and practitioners on identifying risk factors and evidence-based screening of people at high risk of developing CVD (Fig. 1, Table 3). The lack of knowledge about preventable risk factors could hinder the action plans on reducing CVDs [71].

### Study strengths and limitations

Having developed a series of BSEMs, it is possible to examine the associations between a large number of risk factors and risk for total CVD and its different categories. The findings of this study could be of great help for healthcare professionals in recognising strong predictors for applying effective strategies for CVDs prevention. However, this study ignored the possible effects of sleep disorders leading to dyslipidaemia and CVD. Also, the effect of environmental factors, such as air pollution indices, on CVDs incidence, was not taken into account. Moreover, time-varying covariates such as treatments were not considered since this study aimed to evaluate blood factors and lifestyle components.

## Conclusion

The flow of information in indirect and direct paths was revealed by analysing a causal network of risk factors. It also determined predictors and suitable targets for intervention, such as decreasing lipid profile in the general population. Reducing the risk of CVDs could be achieved when multi-factor data is integrated into a complex framework by identifying novel preventable pathways. However, clinical trials are required for lipid profile adjustment as a controllable factor among adults, which could lead to a significant CVD risk reduction.

## Supplementary information

**Additional file 1: **The details of the statistical model and its validation. It includes the following: **Supplementary Material 1.** Modeling description. **Supplementary Material 2.** WinBUGS code BSEM. **Supplementary Table 1.** Factor loading matrix for dietary patterns. **Supplementary Material 3.** An example of Golman Robin test, convergence diagrams, and Monte Carlo error: A. Golman Robin test. B: Convergence diagrams for the first 1000 updates (trace plot). Table C. Monte Carlo error for total CVD

## Data Availability

The datasets used and/or analysed during the current study are available from the corresponding author on reasonable request.

## Abbreviations

CVD: Cardiovascular
ACS: Acute coronary syndrome
BSEM: Bayesian structural equation modeling
NCD: Non-communicable diseases
DM: Diabetes mellitus
BP: Blood pressure
ICS: Isfahan cohort study
AUC: Area under the curve
Tcho: Total cholesterol
HDL-C: *High-density lipoproteins*
LDL-C: Low*-density lipoproteins*
TG: Triglycerides
FBS: Fast blood sugar
HC: Hip criteria
BMI: Body mass index
WC: *Waist* criteria
SBP: Systolic blood pressure
DBP: Diastolic blood pressure
FFQ: Food frequency questionnaire
IPAQ: International physical activity questionnaire
GHQ: General health questionnaire
WHOQOL: World health organisation quality of life
MI: Myocardial infraction
SCD: Sudden cardiac death
UA: Unstable angina
IHD: Ischemic heart disease
SD: Standard deviation
SEM: Structural equation model
MCMC: Markov chain monte carlo
PP *P*-value: Posterior predictive *P*-value
NHVO: Non-hydrogenated vegetable oils
TFA: Trans fatty acids
SFA: Saturated fatty acid
MUFA: Monounsaturated fatty acid
PUFA: Polyunsaturated fatty acid
EMR: Eastern and Mediterrane region
