# Supplementary material for: Pathways leading to prevention of fatal and non-fatal cardiovascular disease: An interaction model on 15 years population-based cohort study
Source: Lipids Health Dis. 2020 Sep 5;19:203. doi: 10.1186/s12944-020-01375-8 (PMC7487611; doi:10.1186/s12944-020-01375-8)
Supplement: Supplementary file 1 — Additional file 1: The details of the statistical model and its validation. It includes the following: Supplementary Material 1. Modeling description. Supplementary Material 2. WinBUGS code BSEM. Supplementary Table 1. Factor loading matrix for dietary patterns. Supplementary Material 3. An example of Golman Robin test, convergence diagrams, and Monte Carlo error: A. Golman Robin test. B: Convergence diagrams for the first 1000 updates (trace plot). Table C. Monte Carlo error for total CVD [file 12944_2020_1375_MOESM1_ESM.docx]

**The details and validation of the statistical model**

**Supplementary Material 1: Modeling description**

A Bayesian inference requires an initial estimate of the unknowns as the prior function. The probability function of observation is then updated. Based on the underlying binary outcomes which follow the normal distribution, let y= (z_i1_, z_i2_, …,z_im_, y_im+1_, y_i2_,…, y_in_) for j=1, …,n, where zij is a dichotomous vector and yij, is a continuous vector. The measurement equation is given by

$y_{i}=\Lambda\omega_{i}+Ad_{i}+\varepsilon_{i} ;$ (1)

In equation (1), $y_{i}$represents the vector p×1 of the observed variables to describe the random vector q×1 of the latent variables $\omega_{i}$, Λ represents the factor load matrix and $\varepsilon_{i}$random vector P×1 of the measurement error. $A$ represents the vector of covariates and $d_{i}$ represents the coefficients vector of covariates. The latent variable $\omega_{i}$ is also considered as $\omega_{i}$ *=*$\left( \begin{matrix} \eta_{i} \\ \xi_{i} \end{matrix} \right)$*.* where $\eta_{i}$ represents the random vector q_1_ × 1 of the latent dependent variables and $\xi_{i}$ represents the random vector *q_2_ × 1 (q_2_ = q-q_1_)* of the independent latent variables.

$$\left( \begin{aligned} \mathrm{total}\mathrm{CVD} \\ HDL-C \\ LDL-C \\ \mathrm{TG} \\ \mathrm{TCho} \\ \mathrm{HC} \\ \mathrm{WC} \\ \mathrm{BMI} \\ depression or anxiety \\ unhealthy diet behavior \\ Smoking Status \\ \mathrm{BP} \\ \mathrm{DM} \\ Environmental Health \\ Social Relationship \\ Mental Health \\ Physical Health \\ Healthy diet \\ Daily physical activity \end{aligned} \right)=\left( \begin{aligned} \lambda_{11} \\ 0 \\ 0 \\ 0 \\ 0 \\ 0 \\ 0 \\ 0 \\ 0 \\ 0 \\ 0 \\ 0 \\ 0 \\ 0 \\ 0 \\ 0 \\ 0 \\ 0 \\ 0 \end{aligned} \begin{aligned} 0 \\ \lambda_{21} \\ \lambda_{22} \\ \lambda_{23} \\ \lambda_{24} \\ 0 \\ 0 \\ 0 \\ 0 \\ 0 \\ 0 \\ 0 \\ 0 \\ 0 \\ 0 \\ 0 \\ 0 \\ 0 \\ 0 \end{aligned} \begin{aligned} 0 \\ 0 \\ 0 \\ 0 \\ 0 \\ \lambda_{31} \\ \lambda_{32} \\ \lambda_{33} \\ 0 \\ 0 \\ 0 \\ 0 \\ 0 \\ 0 \\ 0 \\ 0 \\ 0 \\ 0 \\ 0 \end{aligned} \begin{aligned} 0 \\ 0 \\ 0 \\ 0 \\ 0 \\ 0 \\ 0 \\ 0 \\ \lambda_{41} \\ \lambda_{42} \\ \lambda_{43} \\ 0 \\ 0 \\ 0 \\ 0 \\ 0 \\ 0 \\ 0 \\ 0 \end{aligned} \begin{aligned} 0 \\ 0 \\ 0 \\ 0 \\ 0 \\ 0 \\ 0 \\ 0 \\ 0 \\ 0 \\ 0 \\ \lambda_{51} \\ \lambda_{52} \\ 0 \\ 0 \\ 0 \\ 0 \\ 0 \\ 0 \end{aligned} \begin{aligned} 0 \\ 0 \\ 0 \\ 0 \\ 0 \\ 0 \\ 0 \\ 0 \\ 0 \\ 0 \\ 0 \\ 0 \\ 0 \\ \lambda_{61} \\ \lambda_{62} \\ \lambda_{63} \\ \lambda_{64} \\ 0 \\ 0 \end{aligned} \begin{aligned} 0 \\ 0 \\ 0 \\ 0 \\ 0 \\ 0 \\ 0 \\ 0 \\ 0 \\ 0 \\ 0 \\ 0 \\ 0 \\ 0 \\ 0 \\ 0 \\ 0 \\ \lambda_{71} \\ \lambda_{72} \end{aligned} \right)\times\left( \begin{aligned} \mathrm{total}\mathrm{CVD} \\ \mathrm{Lipids} \\ \mathrm{Anthropometric} \\ Risky behavior \\ \mathrm{Comorbidities} \\ Quality of life \\ Healthy lifestyle component \end{aligned} \right)+\left( \begin{aligned} \mathrm{Age} \\ \mathrm{Sex} \\ Family History of CVD \end{aligned} \right)+\left( \begin{matrix} d_{1} & d_{2} & d_{3} \end{matrix} \right)+\left( \begin{aligned} \varepsilon_{11} \\ \varepsilon_{21} \\ \varepsilon_{22} \\ \varepsilon_{23} \\ \varepsilon_{24} \\ \varepsilon_{31} \\ \varepsilon_{32} \\ \varepsilon_{33} \\ \varepsilon_{41} \\ \varepsilon_{42} \\ \varepsilon_{42} \\ \varepsilon_{51} \\ \varepsilon_{52} \\ \varepsilon_{61} \\ \varepsilon_{62} \\ \varepsilon_{63} \\ \varepsilon_{64} \\ \varepsilon_{71} \\ \varepsilon_{72} \end{aligned} \right) (2)$$

The structural equation is shown in equation 3.

$CVD=\gamma_{1}\times$*Lipids +*$\gamma_{2}\times$ *Anthropometric +*$\gamma_{3}\times$ *Risky behavior +* $\gamma_{4}\times$*Comorbidities +* $\gamma_{5}\times$*Quality of life+*$\gamma_{6}\times$ *Healthy lifestyle component*$+ \delta_{i};$ *(3)*

In equation (3), δ is a random vector q1 × 1 of the residuals. It is also assumed that $\delta_{i}$, and $\varepsilon_{i}$ follow the Normal distribution N [0, Ψε] and N [0, Ψδ].

In the Bayesian approach of structural equation modeling, M is considered as a Model of arbitrary structural equations, θ as the vector of unknown parameters in M, y as observed data, continuous and discrete data with sample size n, and *w* as the latent variables.

The Bayesian estimation of θ as the mean or mode of the posterior function θ | w,y is then performed. Having simulated several observations θ | y, It is possible to obtain averages and other useful statistics through simulated observations. The Markov chain Monte Carlo (MCMC) methods are used to reproduce the observations through their conditional density repeated observations [θ | w, y] and [w | θ, y] get. The Gibbs sampling method is then used.

$$w^{(j)}=(w_{1}^{\left( j \right)},\ldots,w_{b}^{\left( j \right)})$$

$$\theta^{(j)}=(\theta_{1}^{\left( j \right)},\ldots,\theta_{a}^{\left( j \right)})$$

$\theta_{1}^{(j+1)}$ *from* $\left( \theta_{1} | \theta_{2}^{(j)},\ldots,\theta_{a}^{\left( j \right)}, w^{(j)}, y \right),$

$\theta_{2}^{(j+1)}$ *from* $\left( \theta_{2} | \theta_{1}^{(j+1)},\ldots,\theta_{a}^{\left( j \right)}, w^{(j)}, y \right),$

*. . .*

*. . .*

*. . .*

$\theta_{a}^{(j+1)}$*from* $\left( \theta_{a} | \theta_{1}^{(j+1)},\ldots,\theta_{a-1}^{\left( j+1 \right)}, w^{(j)}, y \right),$

$w_{1}^{(j+1)}$ *from* $\left( w_{1} | \theta^{(j+1)},w_{2}^{\left( j \right)},\ldots, w_{b}^{\left( j \right)}, y \right),$

$w_{2}^{(j+1)}$ *from* $\left( w_{2} | \theta^{(j+1)},w_{1}^{\left( j+1 \right)},\ldots, w_{b}^{\left( j \right)}, y \right),$

*. . .*

*. . .*

*. . .*

$w_{b}^{(j+1)}$ *from* $\left( w_{b} | \theta^{(j+1)},w_{1}^{\left( j+1 \right)},\ldots, w_{b-1}^{\left( j+1 \right)}, y \right).$

Finally, the congruent distribution $\left( \theta^{\left( j \right)}w^{\left( j \right)} \right)$ converges to the posterior distribution θ, w | y after the sufficient number of replications.

An essential issue in Bayesian estimation is to consider the prior function for the parameters (1,2). In the next step, the convergence and goodness of fit index of the model are discussed. One of the criteria which was used is the posterior predictive criterion. It compares the data derivatives of the Posterior function (y^rep^) with the observation. If the goodness-of-fit of the model is acceptable, the simulated data under the model should not differ systematically from the observed data. Values close to 0.5 indicate acceptable goodness-of-fit. Two chains and evaluated Goleman-Robin statistics and Monte Carlo error were used to check the convergence of the parameters (3).

**Supplementary Material 2: WinBUGS code BSEM**

model{

for( i in 1 : N ) {

for( j in 1 : 22 ) {

y[i , j] <- yy[i , j]

}

x1[i] <- yy[i , 20]

x2[i] <- yy[i , 21]

x3[i] <- yy[i , 22]

for( j in 1 : 1 ) {

y[i , j] ~ dnorm(mu[i , j], 1)C(y[i , j],y[i , j])

}

for( j in 2 : 19 ) {

y[i , j] ~ dnorm(mu[i , j], psi[j])

ephat[i , j] <- y[i , j] - mu[i , j]

}

mu[i , 1] <- u[1] + beta[1] * x1[i] + ta[1] * x2[i] + eta[i]

mu[i , 2] <- u[2] + beta[2] * x1[i] + ta[2] * x2[i] + xi[i , 1]

mu[i , 3] <- u[3] + beta[3] * x1[i] + ta[3] * x2[i] + lam[1] * xi[i , 1]

mu[i , 4] <- u[4] + beta[4] * x1[i] + ta[4] * x2[i] + lam[2] * xi[i , 1]

mu[i , 5] <- u[5] + beta[5] * x1[i] + ta[5] * x2[i] + lam[3] * xi[i , 1]

mu[i , 6] <- u[6] + beta[6] * x1[i] + ta[6] * x2[i] + xi[i , 2]

mu[i , 7] <- u[7] + beta[7] * x1[i] + ta[7] * x2[i] + lam[4] * xi[i , 2]

mu[i , 8] <- u[8] + beta[8] * x1[i] + ta[8] * x2[i] + lam[5] * xi[i , 2]

mu[i , 9] <- u[9] + beta[9] * x1[i] + ta[9] * x2[i] + xi[i , 3]

mu[i , 10] <- u[10] + beta[10] * x1[i] + ta[10] * x2[i] + lam[6] * xi[i , 3]

mu[i , 11] <- u[11] + beta[11] * x1[i] + ta[11] * x2[i] + lam[7] * xi[i , 3]

mu[i , 12] <- u[12] + beta[12] * x1[i] + ta[12] * x2[i] + xi[i , 4]

mu[i , 13] <- u[13] + beta[13] * x1[i] + ta[13] * x2[i] + lam[8] * xi[i , 4]

mu[i , 14] <- u[14] + beta[14] * x1[i] + ta[14] * x2[i] + xi[i , 5]

mu[i , 15] <- u[15] + beta[15] * x1[i] + ta[15] * x2[i] + lam[9] * xi[i , 5]

mu[i , 16] <- u[16] + beta[16] * x1[i] + ta[16] * x2[i] + lam[10] * xi[i , 5]

mu[i , 17] <- u[17] + beta[17] * x1[i] + ta[17] * x2[i] + lam[11] * xi[i , 5]

mu[i , 18] <- u[18] + beta[18] * x1[i] + ta[18] * x2[i] + xi[i , 6]

mu[i , 19] <- u[19] + beta[19] * x1[i] + ta[19] * x2[i] + lam[12] * xi[i , 6]

xi[i , 1:6] ~ dmnorm(u0[1:6], phi[1:6 , 1:6])

eta[i] ~ dnorm(nu[i], psd)

nu[i] <- ta1[1] * x1[i] + ta1[2] * x2[i] + ta1[3] * x3[i] + gam[4] * xi[i , 1] + gam[5] * xi[i , 2] + gam[6] * xi[i , 3] + gam[7] * xi[i , 4] + gam[8] * xi[i , 5] + gam[9] * xi[i , 6]

dthat[i] <- eta[i] - nu[i]

}

for( j in 1 : 19 ) {

u[j] ~ dnorm(0, 4)

}

lam[1] ~ dnorm(0, 4)

lam[2] ~ dnorm(0, 4)

lam[3] ~ dnorm(0, 4)

lam[4] ~ dnorm(0, 4)

lam[5] ~ dnorm(0, 4)

lam[6] ~ dnorm(0, 4)

lam[7] ~ dnorm(0, 4)

lam[8] ~ dnorm(0, 4)

lam[9] ~ dnorm(0, 4)

lam[10] ~ dnorm(0, 4)

lam[11] ~ dnorm(0, 4)

lam[12] ~ dnorm(0, 4)

gam[1] ~ dnorm(0, psd)

gam[2] ~ dnorm(0, psd)

gam[3] ~ dnorm(0, psd)

gam[4] ~ dnorm(0, psd)

gam[5] ~ dnorm(0, psd)

gam[6] ~ dnorm(0, psd)

gam[7] ~ dnorm(0, psd)

gam[8] ~ dnorm(0, psd)

gam[9] ~ dnorm(0, psd)

for( j in 1 : 19 ) {

psi[j] ~ dgamma(0.01, 0.01)

sigma[j] <- 1 / psi[j]

}

psd ~ dgamma(0.01, 0.01)

sigd <- 1 / psd

phi[1:6 , 1:6] ~ dwish(R[1:6 , 1:6], 8)

phx[1:6 , 1:6] <- inverse(phi[1:6 , 1:6])

ta1[1] <- gam[1]

ta1[2] <- gam[2]

ta1[3] <- gam[3]

for( j in 1 : 6 ) {

gamma[j] <- gam[3 + j]

}

for( j in 1 : 19 ) {

psiepsilon[j] <- sigma[j]

}

psidelta <- sigd

}

**Supplementary Table 1.** Factor loading matrix for dietary patterns

| Food | Western diet | Mediterranean diet | Animal fat diet | Fast food diet |
| --- | --- | --- | --- | --- |
| Fried food | 0.56 | - | - | - |
| Potato | 0.56 | - | - | - |
| Legumes | 0.52 | - | - | - |
| Hydrogenated | 0.51 | -0.44 | - | - |
| Red meat | 0.50 | - | - | - |
| Pickle | 0.45 | - | - | - |
| Rice | 0.45 | - | - | - |
| Non-hydrogenated vegetable oil | - | 0.59 | - | - |
| Fruits | 0.40 | 0.58 | - | - |
| Poultry | - | 0.55 | - | - |
| Fish | - | 0.54 | - | - |
| Vegetables | 0.47 | 0.49 | - | - |
| Olive oil |  | 0.41 | - | - |
| Bread | 0.26 | -0.34 | - | - |
| Cream | - | - | 0.69 | - |
| Whole milk | - | - | 0.59 | - |
| Ghee | - | - | 0.45 | - |
| Butter | 0.22 | - | 0.45 | 0.21 |
| Organ meat | - | - | 0.45 | - |
| Liver | - | - | 0.37 | 0.32 |
| Tallow | - | - | 0.33 | - |
| Dry fruits | 0.25 | - | 0.31 | - |
| Jam | 0.24 | - | 0.28 | 0.25 |
| Soy protein | - | - | - | - |
| Industrial fruit juice | - | - | - | - |
| Hamburger | - | - | - | 0.67 |
| Sausages | - | - | - | 0.66 |
| Pizza | -- | - | - | 0.47 |
| Sweet | - | - | - | 0.46 |
| Carbonated  beverages | - | - | - | 0.42 |
| Nuts | 0.22 | - | - | 0.36 |
| Canned foods | - | - | 0.21 | 0.27 |

**Supplementary Material 3: An example of Golman Robin test, convergence diagrams, and Monte Carlo** **error**

**A. Golman Robin test**

----------------------------80% interval----------------------------

Unnormalized Normalized as plotted

iteration range of pooled mean within of pooled mean within BGR ratio

chains chain chains chain

1051—1100 0.1829 0.1665 0.7949 0.7234 1.099

1101—1200 0.1949 0.1899 0.8468 0.825 1.026

1151—1300 0.2206 0.2212 0.9584 0.9611 0.9972

1201—1400 0.2301 0.2277 1.0 0.9895 1.011

1251—1500 0.2062 0.1926 0.8961 0.8368 1.071

1301—1600 0.1882 0.1714 0.8177 0.7449 1.098

1351—1700 0.1582 0.1464 0.6875 0.636 1.081

1401—1800 0.1688 0.1592 0.7334 0.6919 1.06

1451—1900 0.1783 0.1682 0.7749 0.7311 1.06

1501—2000 0.181 0.1663 0.7864 0.7225 1.088

**B: Convergence diagrams for the first 1000 updates (trace plot)**


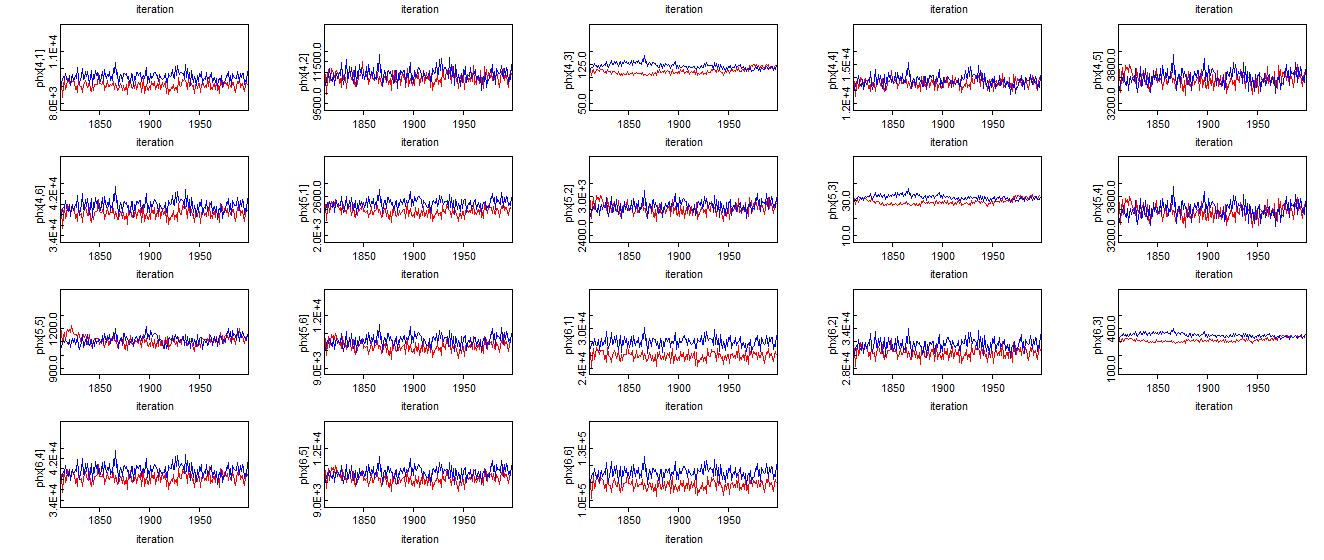


**Table C. Monte Carlo error for total CVD**

| Model | | | |
| --- | --- | --- | --- |
| MC error | | **MC** **error** | |
| Age-> total CVD | 5.30E-05 | **Lipids->** HDL-C  **Lipids->** LDL-C  **Lipids->** Tg  **Lipids->** Cho | -  1.15E-03  1.66E-03  1.91E-03 |
| Family history -> total CVD | 1.40E-03 | **Anthropometric->** HC  **Anthropometric->** WC  **Anthropometric->** BMI | -  1.95E-04  1.12E-04 |
| Sex -> total CVD | 2.04E-03 | **Unhealthy life style->** smoking  **Risky behavior ->**  depression and anxiety  **Risky behavior ->** unhealthy diet behavior | -  5.87E-03  5.58E-03 |
| Lipids -> total CVD | 2.15E-03 | **Comorbidities->** Blood sugar  **Comorbidities->** Blood pressure | -  8.99E-05 |
| Anthropometric -> total CVD | 1.93E-03 | **Quality of life->** Environmental Heath  **Quality of life->** Social Relationship  **Quality of life->** Mental Health  **Quality of life->** Physical Health | -  2.96E-04  2.50E-04  2.76E-04 |
| risky behavior-> total CVD | 4.38E-03 | **Healthy lifestyle component->** physical activity  **Healthy lifestyle component->** Healthy diet behavior | -  9.92E-04 |
| Comorbidities -> total CVD ->Cvd | 2.44E-04 | **Lipids**<-> Anthropometric  **Lipids**<-> Risky behavior  **Lipids**<-> Comorbidities  **Lipids**<-> Quality of life  **Lipids**<->healthy lifestyle component | 8.93E-02  8.18E-02  4.98E-02  7.13E-02  6.79E-02 |
| Quality of life -> total CVD | 1.48E-03 | **Anthropometric**<-> Risky behavior  **Anthropometric**<-> Comorbidities  **Anthropometric**<-> Quality of life  **Anthropometric**<-> healthy lifestyle component | 7.57E-02  4.94E-02  6.93E-02  6.65E-02 |
| Healthy  lifestyle -> total CVD | 1.14E-03 | **Risky behavior** <-> Comorbidities  **Risky behavior** <-> Quality of life  **Risky behavior** <-> healthy lifestyle component | 4.40E-02  6.39E-02  6.13E-02 |
|  |  | **Comorbidities**  <-> Quality of life  **Comorbidities**  <-> healthy lifestyle component | 4.00E-02  4.03E-02 |
|  |  | **Quality of life**<-> healthy lifestyle component | 5.32E-02 |

**References**

1. Lee S-Y, Song X-Y. Basic and advanced Bayesian structural equation modeling: With applications in the medical and behavioral sciences. John Wiley & Sons; 2012.

2. Song X-Y, Lee S-Y. A tutorial on the Bayesian approach for analyzing structural equation models. J Math Psychol [Internet]. 2012;56(3):135–48. Available from: http://www.sciencedirect.com/science/article/pii/S0022249612000065

3. Ntzoufras I. Bayesian modeling using WinBUGS. Vol. 698. John Wiley & Sons; 2011.
